# Congenital pulmonary varix: Two case reports

**DOI:** 10.1097/MD.0000000000028340

**Published:** 2021-12-23

**Authors:** Takuji Maruyama, Shuji Kariya, Miyuki Nakatani, Yasuyuki Ono, Yutaka Ueno, Atsushi Komemushi, Noboru Tanigawa

**Affiliations:** Department of Radiology, Kansai Medical University, 2-5-1 Shinmachi, Hirakata, Osaka, Japan.

**Keywords:** case report, congenital pulmonary varix, pulmonary angiography, pulmonary arteriovenous malformation

## Abstract

**Rationale:**

Patients with congenital pulmonary varix are asymptomatic and require no treatment, but the radiological characteristics of a pulmonary varix are similar to those of a pulmonary arteriovenous malformation, which requires treatment. Pulmonary angiography is useful for obtaining information about the dynamics of pulmonary blood flow to differentiate a pulmonary varix from a pulmonary arteriovenous malformation for the purpose of treatment planning. Two cases of congenital pulmonary varices that were differentiated from pulmonary arteriovenous malformations based on pulmonary angiography findings are presented.

**Patient concerns and diagnosis:**

The first patient was an asymptomatic 39-year-old man. Non-contrast-enhanced computed tomography performed as part of the treatment course for pneumonia showed pulmonary arteriovenous malformation in the right lung. Pulmonary angiography was performed and showed that it was a pulmonary varix. The second patient was an asymptomatic 23-year-old woman. As part of her regular health check-up, she underwent plain chest X-ray examination, which showed an abnormal shadow. Non-contrast-enhanced computed tomography was performed, and pulmonary arteriovenous malformation was suspected. However, contrast-enhanced computed tomography findings suggested that the patient had a congenital pulmonary varix rather than a pulmonary arteriovenous malformation. Pulmonary angiography was subsequently performed for diagnosis, and a pulmonary varix was confirmed.

**Interventions and outcomes:**

No treatment was administered to either patient. The first patient was followed up for four years, and the second patient for two years. Both patients had no symptoms or complications during the follow-up period.

**Lessons:**

Two cases of congenital pulmonary varices were reported. Information about the dynamics of pulmonary blood flow obtained by performing pulmonary angiography was effective in distinguishing between pulmonary arteriovenous malformation and congenital pulmonary varix.

## 1 Introduction

Pulmonary varix refers to the localized dilation of the pulmonary vein that drains into the left atrium.^[1–3]^ It can be categorized into congenital and acquired types, which are characterized by different pathological conditions.^[3–10]^ Congenital pulmonary varix is thought to occur as a result of abnormal development during the transition from splanchnic venous drainage in Carnegie stage XIII to pulmonary venous drainage in stage XVI.^[11]^ Although patients with a congenital pulmonary varix are asymptomatic and do not require any treatment, the radiological characteristics of a pulmonary varix are similar to those of a pulmonary arteriovenous malformation, which requires treatment.^[3,12,13]^ Pulmonary angiography is useful for obtaining information about the dynamics of pulmonary blood flow to differentiate a pulmonary varix from a pulmonary arteriovenous malformation for the purpose of treatment planning.^[3,5,12,14,15]^ Two cases of congenital pulmonary varices that were differentiated from pulmonary arteriovenous malformations based on pulmonary angiography findings are presented.

## 2 Case reports

This case study was approved by our institutional review board, and the requirement to obtain informed consent for inclusion in this study was waived.

### 2.1 Case 1

**Figure 1:**
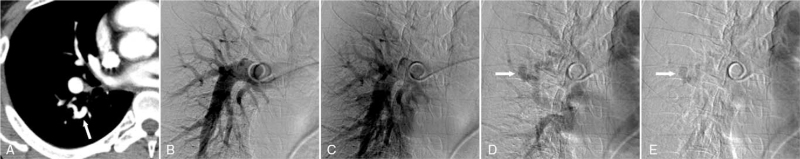
A 39-year-old man. Non-contrast-enhanced computed tomography was performed as part of the treatment course for pneumonia and shows an abnormal shadow in the right lung. (A) Contrast-enhanced computed tomography shows a tortuous vessel in the right lung (white arrow). (B)(C)(D)(E) Digital subtraction right pulmonary angiography. (B)(C) Pulmonary arterial phase and lung parenchymal phase. Abnormal vessels are not identified. (D) Early pulmonary venous phase. A tortuous vessel (white arrow) is seen from the right upper lobe to the lower lobe. (E) Late pulmonary venous phase. Contrast enhancement in the abnormal vessel (white arrow) is persistent.

The patient was a 39-year-old man with no medical history. Non-contrast-enhanced computed tomography, which was performed as part of the treatment course for pneumonia, accidentally showed a pulmonary arteriovenous malformation in the right lung. He was asymptomatic after healing from pneumonia, and physical examination showed no abnormal findings. There were no pulmonary vascular abnormalities in the patient's family history. Contrast-enhanced computed tomography was performed using a multi-detector row scanner (Aquilion, Canon Medical Systems Corporation, Tochigi, Japan). The scanning parameters were as follows: reconstruction thickness, 5 mm; field of view, 330 mm×330 mm; matrix, 512×512; tube voltage, 120 kVp; tube current 108-201 mA. A total of 100 mL of nonionic iodinated contrast material (Iopamidol 300 mg iodine per milliliter (mgI/mL), OYPALOMIN Injection; Fuji Pharma Co., Ltd., Tokyo, Japan) was then administered via an upper limb vein at a flow rate of 3 mL/s. Contrast-enhanced computed tomography revealed a tortuous vessel in the right lung (Fig. 1A). This vessel was seen to be continuous with the pulmonary vein and ran across the lobes from the right upper lobe to the right lower lobe. Although the vessel was not seen to be continuous with the pulmonary artery, pulmonary arteriovenous malformation was suspected as the primary diagnosis, and pulmonary angiography was performed for the purposes of diagnosis and treatment.

Angiography was performed by injecting contrast material (Iopamidol 300 mgI/mL, OYPALOMIN Injection, Fuji Pharma Co., Ltd.) via the right pulmonary artery. In the pulmonary arterial phase and lung parenchymal phase, there were no abnormalities or any indication of the presence of an abnormal vessel that was identified on computed tomography (Fig. 1B, C). In the early pulmonary venous phase, the right pulmonary vein was subsequently identified, and an abnormal vessel was identified simultaneously. The vessel formed the shape of an arc and ran outward from the middle to the lower fields of the right lung (Fig. 1D). The abnormal vessel was connected to the right inferior pulmonary vein, and its contrast enhancement was persistent compared with a normal pulmonary vein in the late pulmonary venous phase. (Fig. 1E). Based on these angiographic findings, the patient was diagnosed with a pulmonary varix instead of a pulmonary arteriovenous malformation. After diagnosis, the patient was followed up for four years without treatment and remained asymptomatic.

On review of the computed tomography images following angiography, the end of the V2 right pulmonary vein was not continuous with the right superior pulmonary vein. Instead, the end of the V2 right pulmonary vein was continuous with the abnormal vein that merged with the V6 right pulmonary vein. The abnormal vein was tortuous, ran within the right lower lobe, and became continuous with the right inferior pulmonary vein. The abnormal vein is considered to be a bypass pathway formed due to a partial defect between the V2 right pulmonary vein and the right superior pulmonary vein. The patient was diagnosed with a congenital pulmonary varix.

### 2.2 Case 2

**Figure 2:**
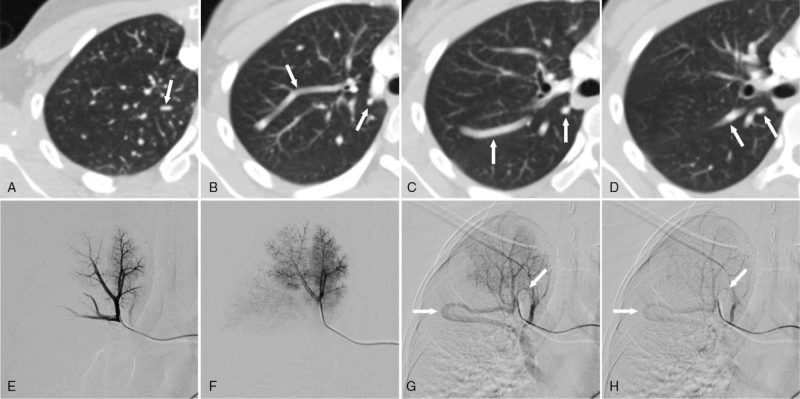
A 23-year-old woman. An abnormal shadow is identified in the right lung. (A)(B)(C)(D) Contrast-enhanced computed tomography shows tortuous vessels that run from the right upper lobe to the lower lobe and are continuous with the right lower pulmonary vein (white arrows). (E)(F)(G)(H) Digital subtraction right pulmonary angiography. (E)(F) Pulmonary arterial phase and lung parenchymal phase. Abnormal vessels are not identified. (G) Early pulmonary venous phase. As normal pulmonary veins are identified, tortuous vessels (white arrows) are also identified. There are two arc-shaped abnormal vessels, which run in and outward from the upper to the middle fields of the right lung. (H) Late pulmonary venous phase. Contrast enhancement in the abnormal vessels (white arrows) is persistent.

The patient was a 23-year-old woman with no medical history. As part of her regular health check-up, she underwent plain chest X-ray examination, which showed an abnormal shadow. She was asymptomatic, and physical examination revealed no abnormal findings. She had no pulmonary vascular abnormalities in her family history. Non-contrast-enhanced computed tomography was performed as an additional examination, and pulmonary arteriovenous malformation was suspected. Contrast-enhanced computed tomography was performed using a multi-detector row scanner (Aquilion PRIME, Canon Medical Systems Corporation). The scanning parameters were as follows: reconstruction thickness, 1 mm; field of view, 300 mm × 300 mm; matrix, 512 × 512; 120 kVp; and 50–600 mA. A total of 80 mL of contrast material (Iopamidol 300 mgI/mL, Iopamiron Inj., Bayer Health Care, Osaka, Japan) was then administered via an upper limb vein at a flow rate of 3.5 mL/sec. Contrast-enhanced computed tomography showed two tortuous vessels in the right lung (Fig. 2A-D). The vessels were seen to be continuous with the ends of the V1 and V2 right pulmonary veins, as well as the right inferior pulmonary vein. The right superior pulmonary vein was partially damaged, and the two abnormal vessels formed bypass pathways from the ends of the V1 and V2 right pulmonary veins. These findings suggest that the patient had a congenital pulmonary varix rather than a pulmonary arteriovenous malformation. Pulmonary angiography was subsequently performed based on the literature evidence suggesting the need for pulmonary angiography for the diagnosis of pulmonary varix.^[16–18]^

Angiography was performed by injecting contrast material (Iopamidol 300 mgI/mL, OYPALOMIN Injection, Fuji Pharma Co., Ltd.) via the right pulmonary artery. The pulmonary arterial phase and lung parenchymal phase appeared normal (Fig. 2E, F). Abnormal vessels identified on computed tomography were not found in the pulmonary arterial phase or lung parenchymal phase. In the early pulmonary venous phase, two tortuous veins were identified, in addition to the right pulmonary vein (Fig. 2G). Both vessels formed the shape of an arc and ran from the upper to the middle fields of the right lung. They were seen to be continuous with the right inferior pulmonary vein, and contrast enhancement was persistent compared with a normal pulmonary vein (Fig. 2H). Angiographic findings suggested that these abnormal vessels were bypass pathways formed because of a partial defect from the ends of V1 and V2 right pulmonary veins to the right upper pulmonary vein. Based on these findings, the patient was diagnosed with a congenital pulmonary varix. After diagnosis, the patient was followed up for two years without treatment and remained asymptomatic.

## 3 Discussion

In 1951, Mouquin et al first reported the use of angiography for the diagnosis of pulmonary varix.^[19]^ Subsequent studies suggested that pulmonary angiography is necessary to distinguish pulmonary varix from pulmonary arteriovenous malformation.^[3,5,12]^ In 1971, Bartram et al published a case series summarizing angiography findings of pulmonary varices based on their patients and other cases reported in previous studies. The findings of this study were used in subsequent studies to establish and modify the diagnostic criteria for pulmonary varix.^[16–18]^ The characteristics of a pulmonary varix on angiography, according to Bartram et al, are as follows^[1]^:

(1) In the arterial phase, there is a normal pulmonary arterial tree without dilatation or capillary shunting.
(2) Since there is no capillary shunting, the vein or veins feeding the varix can be seen in the venous phase, and the varix fills at the same rate as the normal pulmonary veins.
(3) The varix drains directly into the left atrium.
(4) Delayed emptying of the varix compared to the other veins. This results from the large capacity of the varix and its normal rate of filling and emptying.
(5) The varicose appearance and tortuous course affect only the proximal portion of the vein. Peripheral ramifications were normal.

The resolution of computed tomography images was poor at the time of this original study that described the characteristics of pulmonary varices on angiography. In the present cases, the patients were initially examined using contrast-enhanced multi-detector raw computed tomography before pulmonary angiography. While it would have been sufficient to confirm that there was no continuity between the pulmonary artery and vein, it was not possible to achieve this on multi-detector raw computed tomography because the two vessels were close to one another. Thus, pulmonary angiography was necessary to differentiate pulmonary arteriovenous malformation.

In the pulmonary arterial phase, pulmonary arteriovenous malformation is characterized by the presence of a shunt from the pulmonary artery to the pulmonary vein.^[12]^ However, as seen in both of the present cases, patients with a pulmonary varix do not show any abnormalities in the pulmonary arterial phase to the parenchymal phase. Thus, it was possible to easily differentiate pulmonary varix from pulmonary arteriovenous malformation. Although pulmonary angiography is invasive, the present findings are in accordance with the diagnostic criteria and suggest that contrast-enhanced computed tomography alone is not sufficient, and pulmonary angiography is needed to confirm the diagnosis. A contrast-enhanced computed tomography technique such as 4-dimensional computed tomography may be used for the diagnosis of pulmonary varices, because it allows for visualization of the dynamics in the pulmonary arterial and venous phases. However, this technique is not readily available. Thus, pulmonary angiography remains the gold standard for the diagnosis of a congenital pulmonary varix.

Pulmonary varix can be categorized into congenital and acquired types.^[3–10]^ Acquired type is caused by pulmonary hypertension.^[3,4]^ Symptoms such as hemoptysis and complications such as dysphagia and middle lobe syndrome secondary to extrinsic compression can occur because of the acquired type and require treatment.^[4,6]^ Treatment of heart disease that causes pulmonary hypertension is the treatment for the acquired type.^[3]^ On the other hand, congenital pulmonary varix refers to the dilation of a vein that presumably forms as a bypass pathway to compensate for partial congenital damage to the pulmonary vein.^[11]^ Congenital pulmonary varices do not progress, do not cause symptoms or complications, and do not require treatment.^[3]^ Therefore, it is important to differentiate pulmonary varix from pulmonary arteriovenous malformation, which is a disease that requires treatment. In the first case, contrast-enhanced computed tomography images were reviewed after performing pulmonary angiography, and it was possible to identify damage to the pulmonary vein. In the second case, it was possible to identify damage to the pulmonary vein on contrast-enhanced computed tomography before performing pulmonary angiography. Thus, in addition to examining the continuity of abnormal vessels, it would also be helpful in the diagnosis of congenital pulmonary varix to examine computed tomography images for the presence of damage to the pulmonary vein.

## 4 Conclusion

In the present study, two cases of congenital pulmonary varices were reported. Information about the dynamics of pulmonary blood flow in the pulmonary arterial and venous phases should be obtained by performing pulmonary angiography in order to distinguish between pulmonary arteriovenous malformation and congenital pulmonary varix, because the latter does not require any treatment. It would also be useful to identify any damage to the pulmonary vein on computed tomography.

## Author contributions

**Conceptualization:** Shuji Kariya.

**Data curation:** Yasuyuki Ono, Yutaka Ueno.

**Formal analysis:** Yasuyuki Ono.

**Funding acquisition:** Noboru Tanigawa.

**Investigation:** Takuji Maruyama, Shuji Kariya, Miyuki Nakatani, Yasuyuki Ono, Yutaka Ueno, Atsushi Komemushi.

**Methodology:** Takuji Maruyama, Shuji Kariya, Miyuki Nakatani, Yasuyuki Ono, Yutaka Ueno, Atsushi Komemushi.

**Project administration:** Takuji Maruyama, Shuji Kariya, Yutaka Ueno.

**Resources:** Takuji Maruyama, Shuji Kariya.

**Supervision:** Noboru Tanigawa.

**Validation:** Takuji Maruyama, Noboru Tanigawa.

**Visualization:** Takuji Maruyama, Shuji Kariya, Noboru Tanigawa.

**Writing – original draft:** Shuji Kariya.

**Writing – review & editing:** Shuji Kariya.
